# In Vitro Mechanism of Action of *Acanthospermum hispidum* in *Trypanosoma brucei*

**DOI:** 10.1155/2022/1645653

**Published:** 2022-10-18

**Authors:** Aboagye Kwarteng Dofuor, Temitayo Samson Ademolue, Karen Nana Akua Kuampah, Frederick Ayertey, Theresa Manful Gwira

**Affiliations:** ^1^Department of Biological,Physical and Mathematical Sciences, University of Environment and Sustainable Development, Somanya, Ghana; ^2^West African Center for Cell Biology of Infectious Pathogens, University of Ghana, Legon, Accra, Ghana; ^3^Department of Biochemistry,Cell and Molecular Biology, University of Ghana, Legon, Accra, Ghana; ^4^Centre for Plant Medicine Research, Mampong-Akuapem, Mampon, Ghana

## Abstract

African trypanosomiasis is a major neglected tropical disease with significant health and economic concerns in sub-Saharan Africa. In the absence of vaccines for African trypanosomiasis, there is a consideration for alternative sources of chemotherapy. *Acanthospermum hispidum* DC (*A. hispidum*) is a herbal species of the Asteraceae family that is endowed with rich phytochemicals with unknown mechanisms of antitrypanosomal effects. This study aimed to investigate the cellular mechanisms of antitrypanosomal and antioxidant activities of *A. hispidum* against *Trypanosoma brucei (T. brucei)*, a causative protozoan species of African trypanosomiasis. Fractions were prepared from the whole plant of *A. hispidum* through solvent partitioning by employing solvents of varying polarities (hexane, HEX; dichloromethane, DCM; ethyl acetate, EA; aqueous, AQ). The *in vitro* efficacies and mechanisms of antitrypanosomal activities of *A. hispidum* were investigated using a panel of cell biological approaches. GC-MS analysis was used to identify the major compounds with a possible contribution to the trypanocidal effects of *A. hispidum*. *A. hispidum* fractions displayed significant antitrypanosomal activities in terms of half-maximal effective concentrations (EC_50_) and selectivity indices (SI) (AH-HEX, EC_50_ = 2.4 *μ*g/mL, SI = 35.1; AH-DCM, EC_50_ = 2.2 *μ*g/mL, SI = 38.3; AH-EA, EC_50_ = 1.0 *μ*g/mL, SI = 92.8; AH-AQ, EC_50_ = 2.0 *μ*g/mL, SI = 43.8). Fluorescence microscopic analysis showed that at their EC_50_ values, the fractions of *A. hispidum* altered the cell morphology as well as the organization of the mitochondria, nucleus, and kinetoplast in *T. brucei*. At their maximum tested concentrations, the prepared fractions exhibited antioxidant absorbance intensities comparable to the reference antioxidant, Trolox, in contrast to the oxidant intensity of an animal antitrypanosomal drug, diminazene (Trolox, 0.11 A; diminazene, 0.65 A; AH-HEX, 0.20 A, AH-DCM, 0.20 A, AH-EA, 0.13 A, AH-AQ, 0.22 A). GC-MS analysis of the various fractions identified major compounds assignable to the group of alkaloids and esters or amides of aliphatic acids. The results provide useful pharmacological insights into the chemotherapeutic potential of *A. hispidum* toward drug discovery for African trypanosomiasis.

## 1. Introduction

African trypanosomiasis (AT) is a protozoan tsetse-transmitted neglected tropical disease that imposes health and economic concerns on humans and livestock of several sub-Saharan African countries [1, 2]. Chemotherapy is currently the most economically sustainable mode of parasite control due to the absence of vaccines. However, there are challenges of drug resistance and side effects of commercially available drugs. Herbal medicine is consistently gaining attention as an alternative and viable source of AT chemotherapy, and this is evident in the report on antitrypanosomal activities of several plant species in different parts of the world [3–6].

*Acanthospermum hispidum* DC (*A. hispidum*) is an annual herbal plant species that belongs to Asteraceae, a family of flowering plants with numerous species of shrubs, herbs, and trees [7, 8]. The species is native to the South and Central American regions but can also be found in several areas of the African, North American, and Asian continents. Physically, *A. hispidum* is an erect herb of a typical growth height of about 60 cm with oblong, hispid, and gland-dotted leaves of about 1–12 cm in length and 0.5–8 cm in width [7, 8]. The species typically exhibits elliptic pale yellow or glandular-puberulous dark yellow florets with sessile capitula at the branching point of stems or leaf axils while the achenes are enclosed in inner phyllaries with hooked spines [8]. These botanical features may find uses in areas of recreation and gardening aside from their potential nutritional and several potential medicinal and pharmacological properties.

A number of data are available on the characterization of active components of *A. hispidum.* About 26 sesquiterpene lactones were identified in the aerial parts of *A. hispidum* [9]. Terpenoids, alkaloids, glycosides, flavonoids, tannins, and saponins were obtained from the different extracts of *A. hispidum* [10]. Also, polyphenols, together with two flavones, were successfully identified in the leaves of *A. hispidum* [11, 12]. However, studies on the potential connection between phytochemistry and pharmacology of the plant species are scanty. Since the medicinal and traditional uses of plants originate from the combined effects of primary and secondary metabolites [13, 14], the investigation into the mechanisms of phytochemical and pharmacological activities of *A. hispidum* could provide a scientific basis for reported traditional uses.

Traditionally, *A. hispidum* is used in the treatment of jaundice, vomiting, cephalgias, abdominal pain, convulsions, stomachache, constipation, eruptive fever, snake bite, epilepsy, skin ailments, cough, bronchitis, and blennorrhoea [7, 15]. It is used in some parts of South America as a diuretic and as a sudorific. The plant may serve as a useful adjuvant or constituent for antibiotic formulations and antifeedant [7, 15]. *A. hispidum* is also known to possess antidiabetic, antibacterial, anti-inflammatory, antiparasitic, antiviral, antitumor, and antihelmintic properties [7]. Furthermore, nanoparticles of silver and copper oxide prepared from *A. hispidum* were shown to exhibit antimalarial, antimycobacterial, and antifungal activities [16, 17]. However, due to the absence of data on the mechanisms of pharmacological action, there is a poor scientific basis for the reported traditional and medicinal uses of the plant.

Even though antitrypanosomal and antioxidant activities of *A. hispidum* have been reported [18, 19], the corresponding cellular mechanisms of action in trypanosomes have not been investigated. The present study aimed to investigate the phytochemistry of the Ghanaian species of *A. hispidum* for its antitrypanosomal and antioxidant properties in *T. brucei* to provide key insights for AT drug discovery and development. Collectively, the results provide mechanism-based scientific data to support the reported traditional and medicinal properties of *A. hispidum* as far as the treatment of infectious diseases is concerned.

## 2. Materials and Methods

### 2.1. Culture of Parasites and Mammalian Cell Lines

Bloodstream forms of the species *T. brucei* (subspecies *T. brucei brucei* (*T. b. brucei*), strain GUTat 3.1) were cultured *in vitro* to the logarithm phase using Hirumi's Modified Iscove's Medium (HMI9, Thermo Fisher Scientific) with 10% fetal bovine serum (Thermo Fisher Scientific) at 5% CO_2_ and 37°C. Mouse macrophages (RAW 264.7 cell lines) were cultivated *in vitro* to the logarithm phase using Dulbecco's Modified Eagle Media (DMEM, Thermo Fisher Scientific) with 10% fetal bovine serum at 5% CO_2_ and 37°C.

### 2.2. Crude Extraction and Preparation of Fractions

The whole plant of *A. hispidium* was obtained from the arboretum of the Centre for Plant Medicine Research (CPMR), Mampong-Akuapem, Ghana. The plant was authenticated and a voucher specimen with a code of CPMR 5066 was deposited at the herbarium of CPMR. The air-dried whole plant of *A. hispidium* was ground into coarse powder and 198 g of the material was cold-macerated in 2 L of 70% ethanol (V/V) for 72 h to produce the crude extract. The crude extract (HEAH, 15.50 g) was filtered and the ethanol was evaporated off under reduced pressure and temperature using a rotary evaporator (Eyela Co. Ltd, Tokyo, Japan). The residual material was re-extracted twice to ensure exhaustive extraction. The crude extract was partitioned with absolute hexane, absolute dichloromethane, and absolute ethyl acetate (200 mL·×3) to obtain hexane (AH-HEX, 0.93 g), dichloromethane (AH-DCM, 1.23 g), ethyl acetate (AH-EA, 1.40 g) and aqueous fractions (AH-AQ, 9.80 g) respectively. All solvents were absolute. The solvents were evaporated off and the pastes obtained were freeze-dried and stored in a desiccator until further use.

### 2.3. GC-MS Analysis

Gas chromatography-mass spectrometric (GC-MS) analysis was performed using a PerkinElmer GC Clarus 580 Gas Chromatograph interfaced to a mass spectrometer PerkinElmer (Clarus SQ 8S) equipped with Elite-5MS (5% diphenyl/95% dimethyl polysiloxane) fused to a capillary column (*L* ×°I.D. 30 m × 0.25 mm, d*f* 0.25 *μ*m). The following specifications and parameters were used: Oven temperature = 40°C, increase of 3°C/min to 90°C, then 10°C/min to 240°C and held for 15 min at 240°C; Ionization energy = 70 eV; flow rate of helium gas (99.999%) = 1 mL/min; Injection volume = 1 *μ*L; injector temperature = 250°C; ion-source temperature = 150°C; scan interval of mass spectra = 0.1 s; range of fragment size = 45–450 Da; solvent delay = 0 to 2 min; total GC-MS running time = 46 mins; mass-detector = PerkinElmer TurboMass; software for mass spectra and chromatograms = TurboMass version 6.1.0. Interpretation of spectra and identification of compounds was carried out using the database of the National Institute of Standard and Technology (NIST). Samples were dissolved in methanol at a concentration of 50 *μ*g/mL. All solvents used were of the HPLC grade.

### 2.4. Analysis of Cell Viability and Cytotoxicity

Trypanosome cells were seeded at a density of 1.5 × 10^5^ cells/ml in 96-well plates in a two-fold dilution of fractions and incubated for 24 hours. Normal mouse macrophages (RAW 264.7) were seeded at a density of 1.5 × 10^5^ cells/mL for 48 hours before treatment with fractions in a two-fold dilution and subsequent incubation for another 24 hours. Resazurin (10% V/V) was added to the wells and incubated for another 24 hours. Analysis was conducted in quadruplicates. Spectrophotometric absorbance was measured at a wavelength of 570 nm. Diminazene aceturate (Sigma-Aldrich), a known antitrypanosomal drug, was used as a reference drug.

### 2.5. Analysis of Cell Cycle

Trypanosome cells were seeded at a density of 3.0 × 10^5^ cells/ml in a 25 cm^2^ culture dish with fractions for 24 hours and centrifuged at 1700 rpm for 10 mins. Cell pellets were suspended in 1.5 ml of 1x phosphate-buffered saline (PBS) and vortexed well. A 3.5 ml of absolute ethanol was added (final concentration of 70%) to fix cells at −20°C for 1 hour. Cells were centrifuged at 1700 rpm for 10 mins. Cell pellets were suspended with 200 *μ*l of guava cell cycle reagent containing propidium iodide (EMD, Millipore). The distribution of cells at distinct cell cycle phases was measured with the BD LSFortessa X-20 flow cytometer.

### 2.6. Antioxidant Capacity Analysis

The ABTS (2,2′-azino-bis (3-ethylbenzthiazoline-6-sulfonic acid) antioxidant assay kit (Sigma-Aldrich) was used for the analysis of antioxidant capacity with minor modifications. The same procedures as elaborated previously were followed [20]. In summary, *T. b. brucei* cells were seeded at a density of 1.5 × 10^5^ cells/ml on 96-well plates in a two-fold dilution of *A. hispidum* fractions. Myoglobin (20 *μ*l) was added to each well and incubated for 24 hours. ABTS (80 *μ*l) was then added to each well and incubated for approximately 5 mins at room temperature. After inactivating the reaction by adding a stop solution (50 *μ*l), absorbance was read at 405 nm. The final volume of cells and reagents in each well was 200 *μ*l. Trolox ((±)-6-hydroxy-2, 5, 7, 8-tetramethylchromane-2-carboxylic acid) was used as the standard antioxidant. Analysis was performed in duplicates.

### 2.7. Fluorescence Microscopy

Trypanosome cells were treated with fractions at the EC_50_ values for 24 h and centrifuged at 2700 rpm for 10 mins. Cells were resuspended in 1 mL FBS-free HMI9 media and 10 *μ*L of Mito-Tracker Red CMXRos [(1H,5H,11H,15H-xantheno[2,3,4-ij:5,6,7-i′j′]diquinolizin-18-ium,9-[4-(chloromethyl)phenyl]-2,3,6,7,12,13,16,17-octahydro-, chloride)] at 100 *μ*M and incubated for 30 mins. Cells were pelleted at 2700 rpm for 10 mins and resuspended in 1 mL of FBS-free HMI9 media and incubated for another 30 mins. Fixation was performed by incubating cells at 4°C for 1 h in 1 mL of 8% paraformaldehyde in Voorheis modified phosphate-buffered saline (PBS). Washing of cells was carried out by pelleting at 2700 rpm for 10 mins and resuspending in PBS, after which 10–20 *μ*L of cell suspension was spread on poly-L-lysine-coated microscope slides sprayed and wiped clean with 70% ethanol. The slides were allowed to air-dry for 15 mins in a humid chamber and placed in a container with methanol at −20°C for 30 mins. The slides were rinsed in PBS after which 0.1 *μ*g/mL DAPI (4′,6-diamidino-2-phenylindole) was added to the cells. Slides were rinsed again in PBS and 30 *μ*L of mounting media was applied along with coverslips and sealed with nail varnish for observation with the Zeiss Axio Vert.A1 inverted microscope. Data were analyzed with Image J version 2.1.0/1.53c.

### 2.8. Statistical Analysis

Data from cell viability, cytotoxicity, and antioxidant activity assays were analyzed with GraphPad Prism version 5 (Graph Pad Software, San Diego, CA, USA). Histograms from cell cycle data were analyzed using FlowJo V10. The half-maximal effective concentration (EC_50_) was calculated as the concentration that caused a 50% reduction in cell viability. EC_50_ values were calculated from a non-linear regression model using the Hill function. *P* values<0.05 were considered to be significant.

## 3. Results

### 3.1. *A. hispidum* is Selectively Potent against *T. b. brucei*

**Figure 1:**
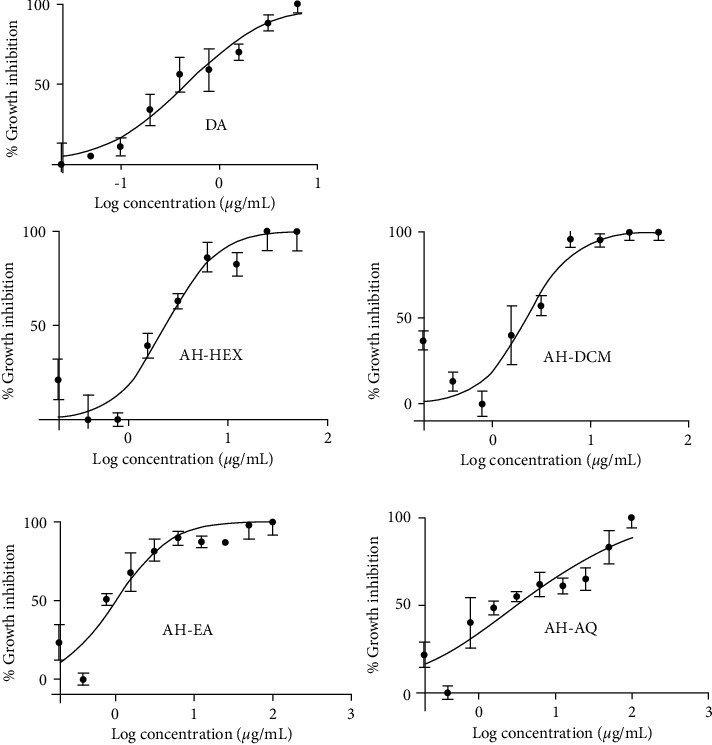
Dose-response curves for fractions of *A. hispidum.* Dose-response curves and half-maximal effective concentration (EC_50_) values were modeled by employing a nonlinear regression of the Hill function. Hexane fraction = AH-HEX, Dichloromethane fraction = AH-DCM, ethyl-acetate fraction = AH-EA, aqueous fraction = AH-AQ, DA = diminazene aceturate.

The fractions of *A. hispidum* were prepared from the crude extract in a solvent partitioning method (Supplementary Figure S1). Solvents were selected based on their varying polarities and partly by virtue of their successful employment in similar bioactivity-guided fractionation analyses [20, 21]. Four major fractions of *A. hispidum* were produced: AH-HEX (hexane), AH-DCM (dichloromethane), AH-EA (ethyl acetate) and AH-AQ (water), in their increasing order of polarity (Supplementary Figure S1). The antitrypanosomal activities of the solvent fractions were determined in a 48-hour cell viability assay. AH-HEX, AH-DCM, AH-EA, and AH-AQ displayed antitrypanosomal potencies of 2.4, 2.2, 1.0, and 2.0 *μ*g/mL, respectively (Figure 1). Also, when normal mouse macrophages (RAW 264.7 cell lines) were treated with the fractions, they exhibited relative selectivities towards *T. brucei* by virtue of approximate selectivity indices of 35.1 (AH-HEX), 38.3 (AH-DCM), 92.8 (AH-EA) and 43.8 (AH-AQ) (Table 1).

### 3.2. *A. hispidum* Alters Morphology and Intracellular Architecture of *T. b. brucei*

**Figure 2:**
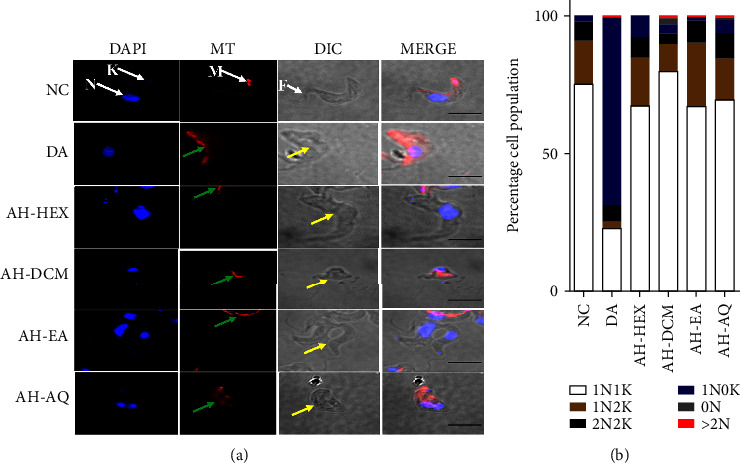
Effects of *A. hispidum* on cell morphology and nucleus-kinetoplast distribution of *T*. *b*. brucei. (a) Parasites were treated with fractions at the EC50 values and investigated using fluorescence microscopy. (b) Percentage cell population of parasites was calculated from an average count of 200 cells in 10 microscopic fields. AH-HEX = hexane fraction, AH-DCM = dichloromethane fraction, AH-EA = ethyl acetate fraction, AH-AQ = aqueous fraction, NC = negative control, MT = mito-tracker red CMXRos, DIC = differential interference contrast, white arrows (*N* = nucleus, *K* = kinetoplast, *M* = mitochondrion, and *F* = flagellum of NC); green arrows = mitochondria of fractions, yellow arrows = cell morphology of fractions, and DA = diminazene aceturate (positive control).

The effects of the fractions on the structure and distribution of trypanosomes were investigated microscopically. The structure of untreated *T. brucei* cells consists of a vermiform shape with tapered ends, a single flagellum, and a ratiometric organization of mitochondrial DNA (kinetoplast, K) and nucleus (N) of 1N1K, 1N2K, or 2N2K, with a ready sequestration of cationic fluorophores across the mitochondrial membrane (Figures 2(a) and 2(b)).

In comparison to untreated cells, treatment with *A. hispidum* fractions resulted in relatively distorted staining of the mitochondrial membrane due to a possible alteration in the sequestration of the mitochondrial membrane potential cationic fluorophore (MitoTracker Red CMXRos) (Figure 2(a)). Collectively, exposure of parasites to *A. hispidum* fractions caused a total loss of kinetoplasts and nuclei in approximately 17% and 3% of observed parasite populations, respectively, even though the induction of multiple nuclei was also evident (Figure 2(b)). Moreover, the fractions induced morphological abnormalities in the cell structure of *T. brucei* (Table 2). Fraction AH-DCM showed the highest morphological aberrations in the trypanosomes (rounded = 5.19%, clumped = 16.56%, shrunken = 13.31% and enlarged = 2.27%) while treatment with AH-AQ showed the least (rounded = 1.49%, clumped = 5.97%, shrunken = 3.73% and enlarged = 0.74%) (Table 2).

### 3.3. *A. hispidum* Alters Cell Cycle Progression in *T. b. brucei*

**Figure 3:**
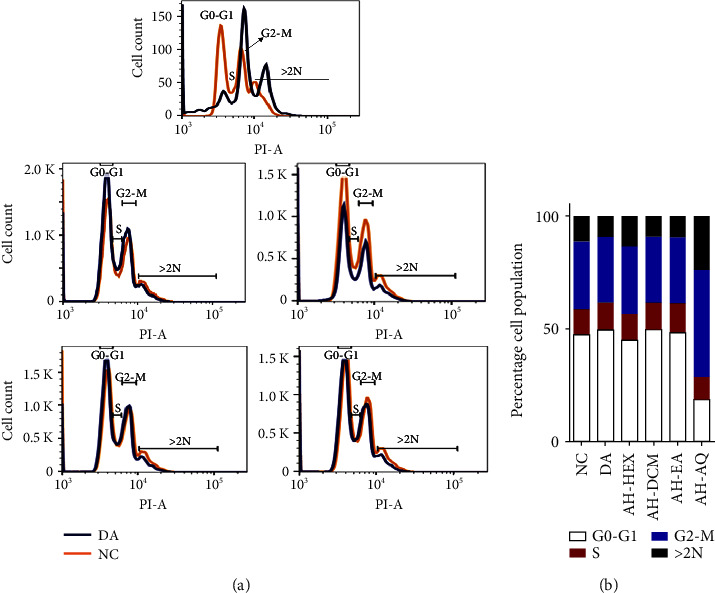
Effects of *A. hispidum* on cell cycle progression of *T.* b. brucei. (a) Progression of cell cycle between untreated parasites and A. hispidum-treated cells. (b) Bar graph of the percentage cell population of parasites for each fraction at distinct cell cycle phases. Cell counts were carried out in triplicates. AH-HEX = hexane fraction, AH-DCM = dichloromethane fraction, AH-EA = ethyl acetate fraction, AH-AQ = aqueous fraction, DA = diminazene aceturate, NC = negative control, and PI = propidium iodide.

*A. hispidum*-treated parasites were also investigated for the effects of fractions on individual cell cycle phases of *T. brucei*. The data indicated differences in the percentage cell population at distinct cell cycle phases and observable differences in the overall cell cycle progression amongst the various fractions (Figure 3, Table S1). AH-DCM induced the highest reduction of percentage cell cycle population of approximately 46% and 12% at G0-G1 and S phases, respectively (Figure 3(b), Table S1). The same fraction also resulted in the highest number of multiple nuclei with a percentage parasite population of about 13% (Figure 3(b), Table S1).

### 3.4. *A. hispidum* Exhibits Antioxidant Activity in *T. b. brucei*

**Figure 4:**
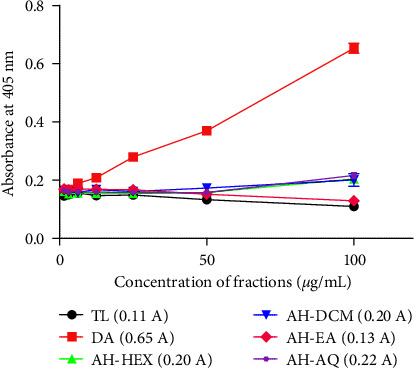
Antioxidant activities of fractions in *T. b brucei*. Absorbance was read within a concentration range of 1.5625 and 100 *μ*g/mL. Hexane fraction = AH-HEX, dichloromethane fraction = AH-DCM, ethyl-acetate fraction = AH-EA, aqueous fraction = AH-AQ, DA = Diminazene aceturate, TL = Trolox, *A* = absorbance units at 100 *μ*g/mL.

The antioxidant properties of fractions in the parasites were explored by utilizing the reducing properties of ABTS (2,2′-azino-bis (3-ethylbenzthiazoline-6-sulfonic acid) and the antioxidant potential of the water-soluble analog of vitamin E (Trolox), as previously reported [20]. A dose-dependent increase in absorbance of an antitrypanosomal in *T. brucei* is a strong indication of its oxidant capacity [20]. This was the case for the animal African antitrypanosomal (AAT) drug diminazene, which exhibited a strong oxidant potential (Figure 4). In contrast to diminazene, *A. hispidum* fractions exhibited dose-dependent trends in absorbance intensities similar to that of Trolox (Figure 4). At the maximum tested concentration of 100 *μ*g/mL, the fractions displayed absorbance intensities of 0.2, 0.2, 0.13, and 0.22 A for AH-HEX, AH-DCM, AH-EA, and AH-AQ, respectively (Figure 4).

### 3.5. GC-MS Analysis of *A. hispidum*

**Figure 5:**
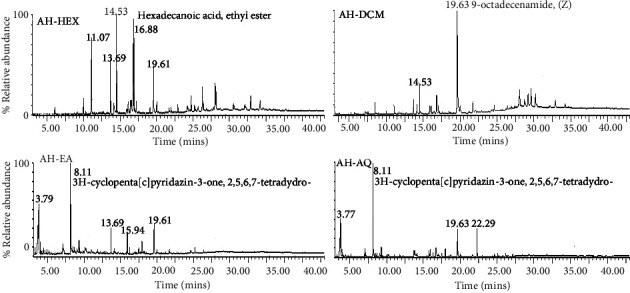
GC-MS fingerprint of *A. hispidum*. The most abundant secondary metabolites were identified as ethyl hexadecanoate (AH-HEX), 9(Z)-octadecenamide (AH-DCM), and 2,5,6,7-tetrahydro-3H-cyclopenta[c]pyridazin-3-one (AH-EA/AH-AQ), with retention times of approximately 14.53, 19.63 and 8.11 mins, respectively. AH-HEX = hexane fraction, AH-DCM = dichloromethane fraction, AH-EA = ethyl acetate fraction, AH-AQ = aqueous fraction.

In order to determine phytochemicals with possible roles in the antitrypanosomal activities, *A. hispidum* was elucidated in qualitative GC-MS analysis. The most abundant compounds identified in the fractions were ethyl hexadecanoate (AH-HEX), 9(Z)-octadecenamide (AH-DCM), and 2,5,6,7-tetrahydro-3H-cyclopenta[c]pyridazine-3-one (AH-EA/AH-AQ) with retention times of approximately 14.53 (*m/z* = 284.4), 19.63 (*m/z* = 281.4), and 8.11 (*m/z* = 136.1) mins, respectively (Figures 5, S2–S4). Thus, GC-MS analysis of *A. hispidum* identified major compounds assignable to the group of alkaloids and esters or amides of aliphatic acids.

## 4. Discussion

Several phytochemicals such as terpenoids, alkaloids, glycosides, flavonoids, and saponins have been identified and characterized in *A. hispidum* [7]. The plant species have also been linked to a myriad of pharmacological and medicinal properties [7, 18, 19]. However, there is no reported investigation linking the phytochemistry of *A. hispidum* to its pharmacological effects against microbial infections with regard to cellular mechanisms of action. The present study sought to bridge this gap in the context of antitrypanosomal and antioxidant efficacies of *A. hispidum* in *T. brucei.*

We observed growth inhibitory concentrations ranging from 1–2.4 *μ*g/mL for the trypanosome-treated cells, which was comparable to diminazene, an AAT drug often used to treat the disease. Moreover, the selectivity index (SI) obtained for the fractions ranged from 35.1–92.8 compared to 123.5 for diminazene. Collectively, these are indicative of good potency and selectivity with minimal toxicity of the fractions against trypanosomes *in vitro*. Since diminazene is known to exhibit considerable toxicity, *A. hispidum* thus offers an alternative and promising chemotherapeutic potential in the context of drug discovery and development for African trypanosomes.

In trypanosomes, the beginning and completion of kinetoplast replication usually precede that of the nucleus, with replication being completed by the end of the G2 phase [22]. In comparison to untreated cells, *A. hispidum* fractions induced multiple nuclei, kinetoplastid, and corresponding morphological alterations in *T. brucei*. These observations may point to a possible relationship between the ratiometric pattern of nucleus-kinetoplast and morphology in the context of cell cycle progression which might provide insights into the antitrypanosomal effects of *A. hispidum*.

Mitochondrial membrane potential is known to regulate the matrix configuration and cytochrome *c* release during apoptosis [23]. Moreover, the release of cytochrome *c* from the outer mitochondrial membrane is triggered by a host of interacting proteins during the intrinsic pathway of apoptosis [24–27]. Thus, the observed comparative alteration in the morphology of the mitochondrial membrane in CMXRos-stained cells between treated and untreated parasites may suggest a shift in the mitochondrial membrane potential and a subsequent induction of apoptosis-like cell death by *A. hispidum*. However, this study does not provide direct confirmation of the membrane potential and, thus, further studies that demonstrate a change in mitochondrial membrane potential as a mode of antitrypanosomal action of *A. hispidum* would further underscore its pharmacological potential.

The induction of oxidative stress may represent an important mechanism of action for a number of potent antitrypanosomals. These include synthetic commercially available antitrypanosomal drugs such as nifurtimox and diminazene, as well as natural plant secondary metabolites [20, 28]. The critical role played by oxidative stress in the progression of Chagas disease suggests that antioxidants may also act to regulate the growth of trypanosomes [29]. Indeed, natural antioxidants have been proposed as adjuvants for the treatment of Chagas disease [29]. Thus the observed antioxidant properties of *A. hispidum* may serve to supplement its antitrypanosomal properties.

In the present study, secondary metabolites with previously unknown antitrypanosomal properties were identified. This contributes towards finding alternative sources of antitrypanosomal chemotherapy as well as provides potential insights into new phytochemical-pharmacological associations of *A. hispidum*. It also enriches the stock and variety of trypanocidal phytochemicals as obtainable from different plant species [20, 21, 30]. However, a confirmation of the antitrypanosomal and antioxidant properties of the identified compounds is critical. Future studies may thus focus on the isolation and subsequent antitrypanosomal and antioxidant characterization of the major compounds [ethyl hexadecanoate, 9(Z)-octadecenamide and 2, 5, 6, 7-tetrahydro-3H-cyclopenta[c]pyridazin-3-one], with possible extension to minor ones in order to investigate potential synergistic interactions of *A. hispidum* phytochemistry. This would inform the discovery and development of new antitrypanosomal drugs from *A. hispidum*.

## Figures and Tables

**Table 1:** Antitrypanosomal activities of fractions.

Fractions	Mean EC_50_ (*μ*g/mL) ± SEM		SI
	*T. b. brucei*	RAW 264.7	
AH-HEX	2.4 ± 0.06	84.3 ± 1.1	35.1
AH-DCM	2.2 ± 0.06	68.9 ± 0.9	38.3
AH-EA	1.0 ± 0.06	92.8 ± 1.0	92.8
AH-AQ	2.0 ± 0.10	87.5 ± 1.0	43.8
DA	0.6 ± 0.09	74.1 ± 1.1	123.5

SI (selectivity index) was estimated as the ratio of the EC_50_ value in RAW 264.7 cell lines to the EC_50_ value in *T. b. brucei*. High SI values denote greater selectivity towards *T. b. brucei* in the presence of RAW 264.7 cell lines. Standard errors of the mean (SEM) were calculated from quadruplicate sets of EC_50_ values. Hexane fraction = AH-HEX, dichloromethane fraction = AH-DCM, ethyl-acetate fraction = AH-EA, aqueous fraction = AH-AQ, DA = Diminazene aceturate.

**Table 2:** Percentage cell distortion of *T. b. brucei* treated with *A. hispidum* fractions.

Fractions	Percentage cell population				
	Rounded	Clumped	Shrunken	Enlarged	Normal
NC	0	5.78	0	0	91.34
DA	4.96	3.31	0.83	14.88	20.66
AH-HEX	0.95	2.86	6.19	3.81	83.33
AH-DCM	5.19	16.56	13.31	2.27	54.87
AH-EA	0.71	15.13	2.13	0.71	76.83
AH-AQ	1.49	5.97	3.73	0.74	83.95

AH-HEX = hexane fraction; AH-DCM = dichloromethane fraction; AH-EA = ethyl acetate fraction; AH-AQ = aqueous fraction; DA = diminazene aceturate, NC = negative control.

## Data Availability

All the data supporting the study have been provided in the manuscript.

## Abbreviations

AT: African trypanosomiasis
*T. brucei*: *Trypanosoma brucei*
*A. hispidum*: *Acanthospermum hispidum* DC.

## Supplementary Materials

Supplementary Materials Figure S1: Crude extraction and preparation of fractions. AH-HEX = hexane fraction, AH-DCM = dichloromethane fraction, AH-EA = ethyl acetate fraction, AH-AQ = aqueous fraction, and HEAH = crude extract. Figure S2: Mass fragmentation for hexadecanoic acid and ethyl ester (*m/z* = 284.4). Ethyl hexadecanoate was identified through GC-MS analysis of hexane fraction of *A. hispidum.* Figure S3: Mass fragmentation for 9-octadecenamide and Z-(*m/z* = 281.4). 9(Z)-octadecenamide was identified through GC-MS analysis of dichloromethane fraction of *A. hispidum*. Figure S4: Mass fragmentation for 2,5,6,7-tetradydro-3H-cyclopenta[c]pyridazin-3-one (*m/z* = 136.1). 2,5,6,7-tetradydro-3H-cyclopenta[c]pyridazin-3-one was identified through GC-MS analysis of ethylacetate and aqueous fractions of *A. hispidum*. Table S1: Percentage cell population in cell cycle phases. AH-HEX = hexane fraction, AH-DCM = dichloromethane fraction, AH-EA = ethyl acetate fraction, AH-AQ = aqueous fraction, DA = diminazene aceturate, NC = negative control.
