# Supplementary material for: In Vitro Mechanism of Action of Acanthospermum hispidum in Trypanosoma brucei
Source: Adv Pharmacol Pharm Sci. 2022 Oct 18;2022:1645653. doi: 10.1155/2022/1645653 (PMC9596247; doi:10.1155/2022/1645653)
Supplement: Supplementary Materials — Figure S1: Crude extraction and preparation of fractions. AH-HEX = hexane fraction, AH-DCM = dichloromethane fraction, AH-EA = ethyl acetate fraction, AH-AQ = aqueous fraction, and HEAH = crude extract. Figure S2: Mass fragmentation for hexadecanoic acid and ethyl ester (m/z = 284.4). Ethyl hexadecanoate was identified through GC-MS analysis of hexane fraction of A. hispidum. Figure S3: Mass fragmentation for 9-octadecenamide and Z-(m/z = 281.4). 9(Z)-octadecenamide was identified through GC-MS analysis of dichloromethane fraction of A. hispidum. Figure S4: Mass fragmentation for 2,5,6,7-tetradydro-3H-cyclopenta[c]pyridazin-3-one (m/z = 136.1). 2,5,6,7-tetradydro-3H-cyclopenta[c]pyridazin-3-one was identified through GC-MS analysis of ethylacetate and aqueous fractions of A. hispidum. Table S1: Percentage cell population in cell cycle phases. AH-HEX = hexane fraction, AH-DCM = dichloromethane fraction, AH-EA = ethyl acetate fraction, AH-AQ = aqueous fraction, DA = diminazene aceturate, NC = negative control. [file 1645653.f1.pdf]

Figure S1: Crude extraction and preparation of fractions

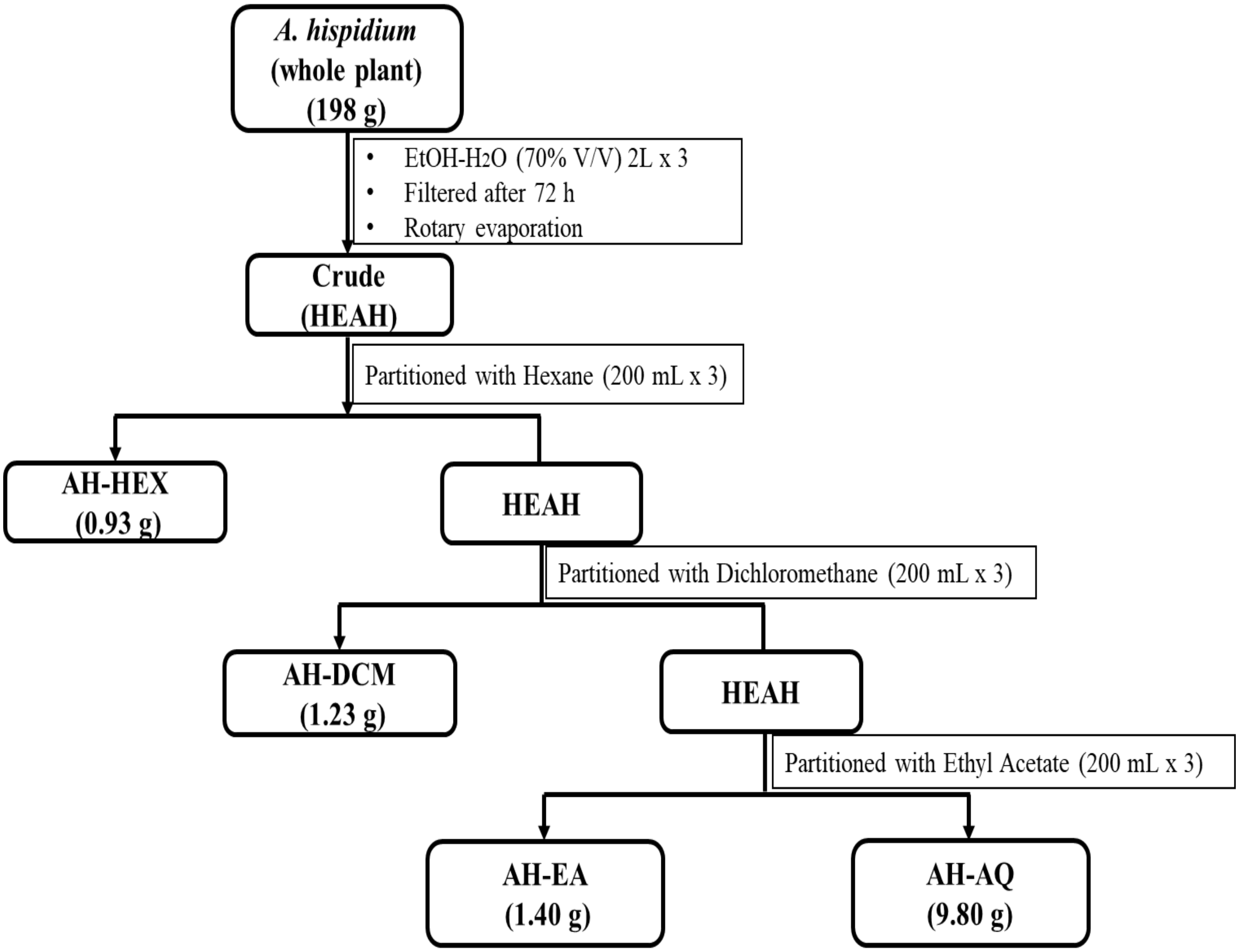

AH-HEX=hexane fraction, AH-DCM=dichloromethane fraction, AH-EA=ethyl acetate fraction, AH-AQ=aqueous fraction, HEAH=crude extract

.

Figure S2: Mass fragmentation for hexadecanoic acid, ethyl ester (*m/z*=284.4)

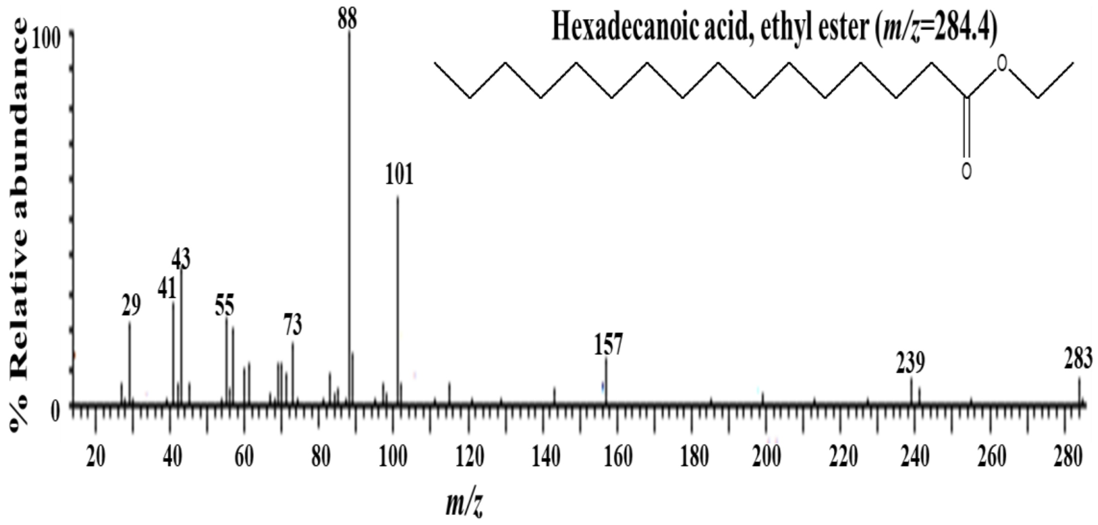

Ethyl hexadecanoate was identified through GC-MS analysis of hexane fraction of *A. hispidum*

Figure S3: Mass fragmentation for 9-octadecenamide, Z-(*m/z*=281.4)

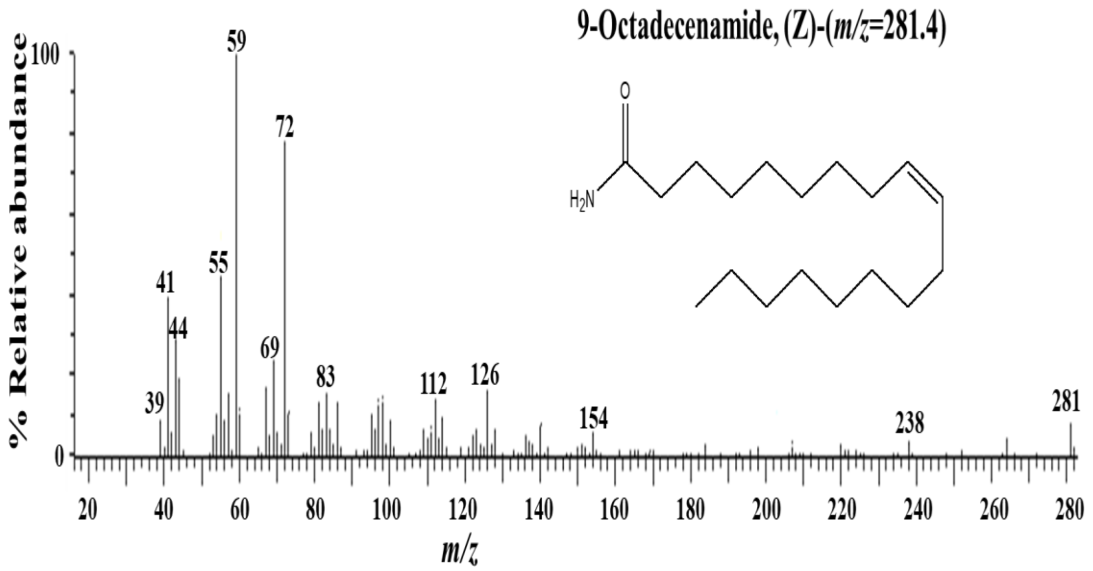

9(Z)-octadecenamide was identified through GC-MS analysis of dichloromethane fraction of *A. hispidum*

Figure S4: Mass fragmentation for 2,5,6,7-tetradydro-3H-cyclopenta[c]pyridazin-3-one (m/z=136.1)

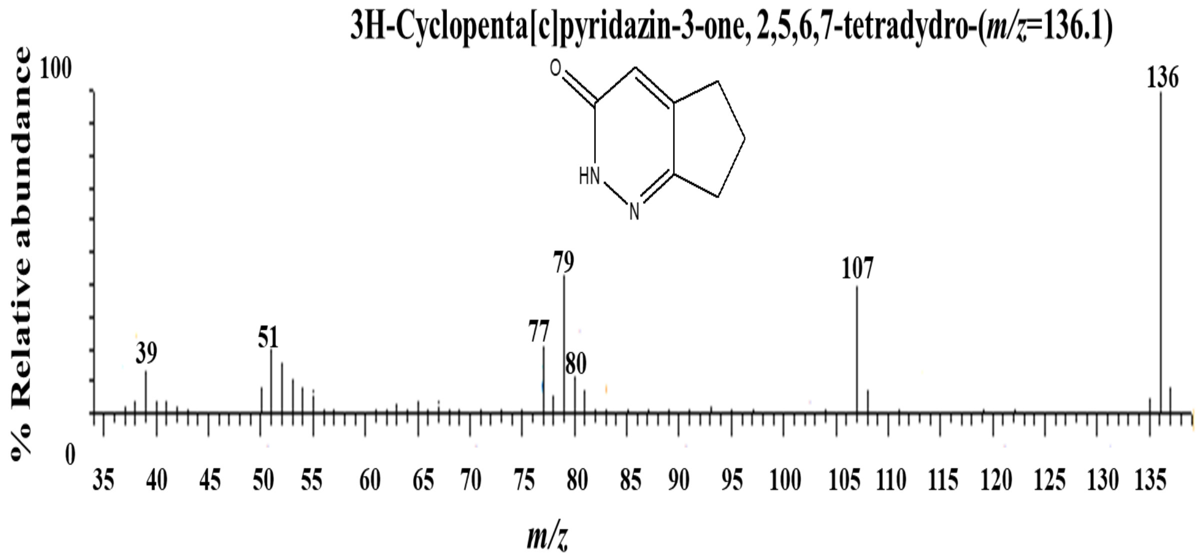

2,5,6,7-tetradydro-3H-cyclopenta[c]pyridazin-3-one was identified through GC-MS analysis of ethylacetate and aqueous fractions of *A. hispidum*

Table S1: Percentage cell population in cell cycle phases

	Percentage cell cycle population			
**Fractions**	**G0-G1**	**S**	**G2-M**	**>2N**
NC	47.19	11.76	30.05	10.99
DA	18.32	10.33	47.43	23.91
AH-HEX	48.80	12.33	29.29	8.8
AH-DCM	45.44	11.45	30.05	12.67
AH-EA	49.75	11.85	29.47	8.93
AH-AQ	48.53	12.77	29.49	9.2

AH-HEX=hexane fraction, AH-DCM=dichloromethane fraction, AH-EA=ethyl acetate fraction, AH-AQ=aqueous fraction, DA=diminazene aceturate, NC=negative control
